# Anisotropy of Mechanical Properties of 3D-Printed Materials—Influence of Application Time of Subsequent Layers

**DOI:** 10.3390/ma18163845

**Published:** 2025-08-15

**Authors:** Marcin Maroszek, Izabela Hager, Katarzyna Mróz, Mateusz Sitarz, Marek Hebda

**Affiliations:** 1 Department of Materials Engineering, Faculty of Materials Engineering and Physics, Cracow University of Technology, Warszawska 24, 31-155 Kraków, Poland; marek.hebda@pk.edu.pl; 2 Chair of Building Materials Engineering, Faculty of Civil Engineering, Cracow University of Technology, Warszawska 24, 31-155 Kraków, Poland; izabela.hager@pk.edu.pl (I.H.); katarzyna.mroz@pk.edu.pl (K.M.); mateusz.sitarz@pk.edu.pl (M.S.)

**Keywords:** additive manufacturing, anisotropy of mechanical properties, building materials, 3D printing, interlayer bond strength, print parameters

## Abstract

Three-dimensional concrete printing (3DCP) is an emerging additive manufacturing technology with increasing application potential in the construction industry, offering advantages such as reduced labor requirements, shortened construction time, and material efficiency. However, structural integrity remains a challenge, particularly due to weak interlayer bonding resulting from the layered manufacturing process. This study investigates the mechanical performance and anisotropy of 3D-printed mineral-based composites with respect to the time interval between successive layers. Specimens were printed with varying interlayer intervals (0, 25, and 50 min) and tested in different loading directions. Flexural, compressive, and tensile strengths (direct and splitting methods) were measured both parallel and perpendicular to the layer orientation. Results showed a clear degradation in mechanical properties with increasing interlayer time, particularly in the direction perpendicular to the layers. Flexural strength decreased by over 25% and direct tensile strength by up to 40% with a 25 min interval. Compressive strength also declined, though less dramatically. Compared to cast specimens, printed elements showed 3–4 times lower compressive strength, highlighting the significant impact of interlayer cohesion. This study confirms that both the time between layers and the loading direction strongly influence mechanical behavior, underlining the anisotropic nature of 3DCP elements and the need for process optimization to ensure structural reliability.

## 1. Introduction

Three-dimensional concrete printing (3DCP) is an example of additive manufacturing (AM) technology, based on the layer-by-layer deposition of material to form three-dimensional elements. Its potential for production automation is increasingly applied across various technical sectors, including construction, where it is used for manufacturing prefabricated elements as well as building components directly on-site [1,2,3,4]. The rapid growth of the global construction industry brings new challenges that drive the development of 3D printing technologies. The application of this technology contributes to shorter construction times and reduces the need for manual labor [5]. It also enables the realization of structures that would be impossible or economically unfeasible to execute using conventional construction methods [6]. Equally important is the opportunity for sustainable construction through the implementation of resource-efficient technologies that still ensure high material and structural quality. Three-dimensional printing supports this goal by facilitating the practical use of composite cements and geopolymer materials, which help reduce reliance on energy-intensive Portland cement [7,8,9]. Moreover, 3D printers allow for the use of extrusion-based forming techniques for the faster and automated construction of objects, including those with complex geometries, while minimizing material usage and labor demands [10,11,12].

The use of this method for producing building elements presents numerous challenges. A fundamental issue is the selection of a material with appropriate rheological properties in the fresh state and adequate mechanical performance after setting. To achieve this, the use of suitable admixtures for setting time control and plasticization is essential [13,14,15]. Mixture design is based on an experimental approach, taking into account parameters such as workability, pumpability, open time, and extrudability. Proper flowability is critical for the extrusion process while maintaining the geometric stability of successive layers. Excessive fluidity may lead to the buckling or collapse of the structure, whereas an overly stiff mixture reduces interlayer adhesion. Rheology is therefore one of the key factors determining the success of the printing process [1,16,17,18].

Process parameters are equally important, as they directly affect extrusion stability and, consequently, print quality [19]. In order for 3DCP technology to be applied in the production of structural elements, standardization is required [20,21]. Components produced through 3D printing must demonstrate high repeatability, quality, and the absence of defects that could compromise structural integrity [22]. Another critical issue is the bond between consecutively deposited layers, which often represents the weakest point in the printed element [23].

**Figure 1:**
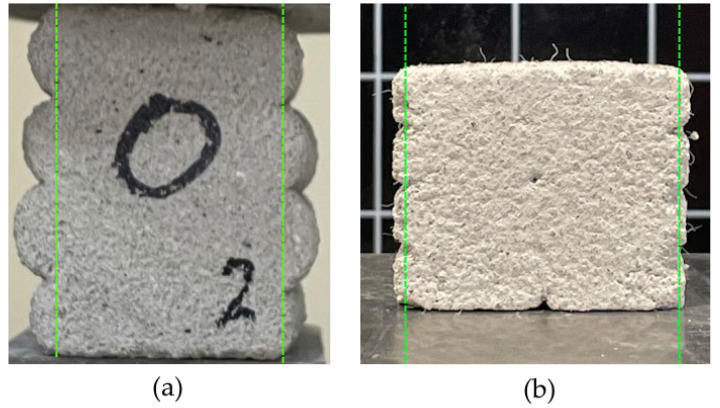
The geometry of printouts and the interlayer bond: (**a**) with round edge; (**b**) rectangular cross-section. Green lines indicate the extent of the interlayer bond in the presented 3D printed specimens.

Several factors influence the interlayer bond strength in 3D-printed structures made from mineral-based materials. One such factor is the cross-sectional shape of the printed path. To achieve optimal interlayer bonding, it is recommended to aim for a rectangular cross-section, as it provides the largest contact surface for transferring forces between layers (Figure 1). Another factor affecting layer cohesion is the nozzle offset distance. As this distance increases, the interlayer bond strength decreases, and more voids tend to form between layers, further weakening the connection [24,25,26].

**Figure 2:**
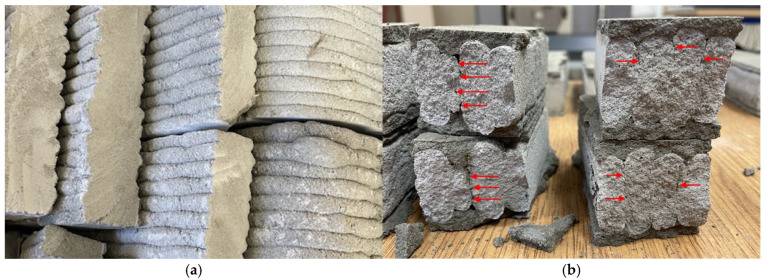
(**a**) Structure of 3D-printed concrete elements; (**b**) defects in the interlayer bond. Red arrows highlight discontinuities at the interlayer interfaces.

The time interval between the deposition of successive layers also plays a critical role. A longer delay, particularly when using fast-setting mixtures, may result in the formation of numerous voids, significantly reducing the structural integrity of the print. These voids are more prevalent in structures where the time gap between layers is extended. This is primarily due to surface moisture loss (cold joint), which occurs as the interval increases, leading to a reduction in bond strength (Figure 2) [23,27,28,29,30]. The interlayer time interval is determined by both the geometry of the printed object and the printing speed. For small prefabricated elements, the interval may be limited to a few minutes. However, when printing larger building components with significant surface areas, the time between layers can extend to approximately one hour. In such cases, it may be necessary to divide the geometry of the printed element to avoid excessive delays between layer deposition, which could compromise the structural integrity of the printed object.

Research teams working in this area have applied various time intervals in their experimental studies. Pan et al. introduced layer deposition intervals of 20 s, 15, 30, 45, and 60 min, with results clearly indicating a reduction in interlayer bond strength as measured by direct tensile testing [31]. Similar outcomes were reported by Chen et al. and Tay et al., who applied time intervals of 20 s, 1, and 10 min, and 1, 5, 10, and 20 min, respectively [28,32].

Hardened 3D-printed material exhibits anisotropic behavior, meaning that its properties vary depending on the direction of observation. In the case of 3D-printed structures produced using additive manufacturing technologies, anisotropy results from the layer-by-layer nature of the printing process.

Anisotropy is considered one of the main drawbacks of structures fabricated via 3D printing, which necessitates thorough investigation of their mechanical properties [25,27,30]. Researchers adopt various approaches to material testing. The most commonly conducted strength tests on 3D-printed specimens include tensile testing, although compressive strength and three-point bending tests are also widely performed [28,29,31,33,34].

Three-dimensional concrete printing technology is a rapidly evolving field entering the construction sector. The diversity of printing techniques and process parameters offers vast potential, particularly in the individualized fabrication of structural elements, yet it also presents numerous challenges in terms of standardization and market implementation. Despite the numerous studies cited earlier, there is still no standardized method or clear guidelines for assessing the quality of 3D-printed elements, highlighting the need for a reliable testing procedure to evaluate the adhesion quality between layers in printed materials. This is likely due to the relatively short time that 3D printing has been in use, especially within the construction industry. However, the standardization of quality verification methods is essential for the full-scale adoption of 3D printing in construction.

The aim of this study was to compare various mechanical testing methods for elements printed in different orientations, with a focus on evaluating interlayer bond quality under conditions simulating large-scale printing. The conducted review of testing procedures revealed several viable approaches.

## 2. Materials and Methods

One of the key challenges in 3D printing technology is ensuring sufficient interlayer bond strength, which significantly determines the mechanical performance of the final structures. Due to the characteristic layer-by-layer deposition of material, mechanical properties exhibit pronounced anisotropy—they are highly dependent on the orientation relative to the build axis. Particularly critical are the interfaces between layers, where so-called cold joints often form, serving as potential points of damage initiation.

As a result, the measurement of interlayer adhesion strength and in-depth analysis of the mechanisms governing the formation and degradation of these bonds represent a major research direction in the study of anisotropy in printed structures. A review of the literature confirms that process parameters, such as the time gap between layer depositions, nozzle speed, and nozzle height, have a significant impact on the strength of interlayer bonds in 3D-printed elements [35,36,37]. The standardization of testing methods and the development of predictive models relating process parameters to bond quality are essential for further industrialization of the technology. Understanding these phenomena is crucial not only for improving structural integrity but also for minimizing the directional variability of mechanical properties [38,39].

**Figure 3:**
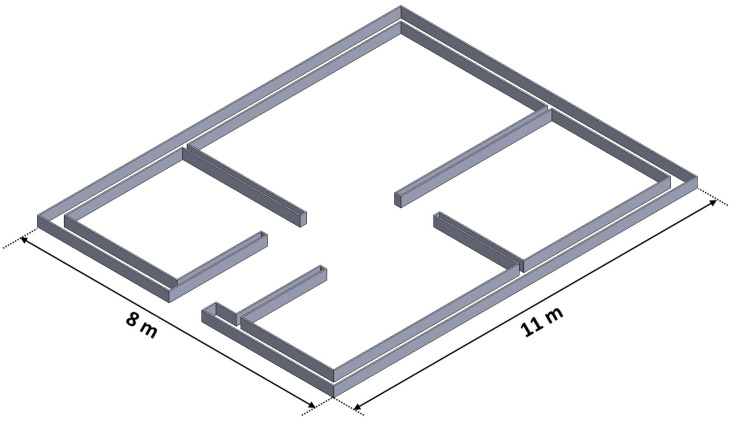
Schematic outline of building walls for estimating the time interval between layers.

As a reference for introducing time intervals between the layers of printed specimens, an estimation was made based on the 3D printing process of a building with a usable floor area of approximately 80 m^2^ (Figure 3). Based on the parameters of the available printing equipment, the maximum printing speeds for 20 mm and 40 mm nozzles were determined to be 100 mm/s and 50 mm/s, respectively. 

Assuming the use of a 40 mm nozzle for printing a single perimeter contour of the walls with standard infill providing structural stability, the path length for one layer is approximately 150 m. With a printing speed of 50 mm/s, the estimated time to complete a single layer is about 50 min, which defines the maximum allowable time interval between successive layers of deposited material. The reference sample was printed without any time gap between layers, while an intermediate variant was printed with a 25 min interval between successive layers.

### 2.1. Methods

Due to the absence of standardized testing methods specifically dedicated to 3D-printed concrete specimens for determining mechanical properties and interlayer bond quality, experimental procedures are typically based on standards developed for cast concrete. The methods proposed by researchers for evaluating the strength parameters of printed structures include compressive strength, direct tensile strength, splitting tensile strength, and three-point bending.

**Figure 4:**
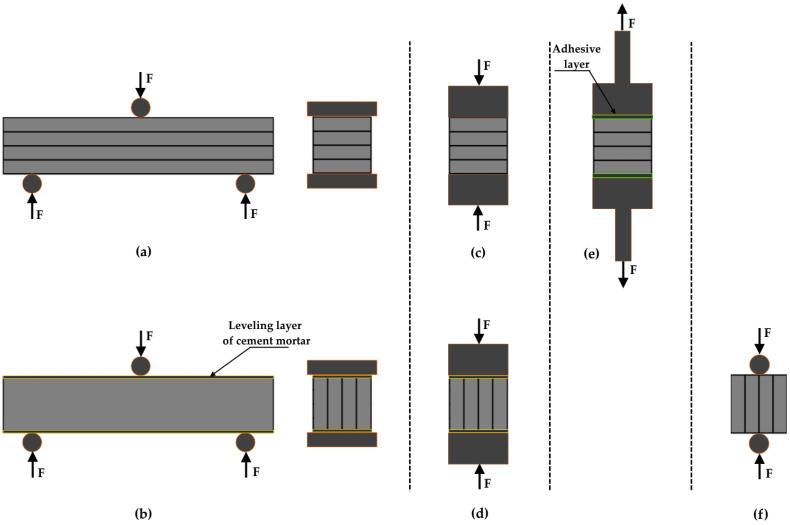
Schemes of strength test for 3D prints: (**a**) three-point bending test, perpendicular to the layer–interface plane; (**b**) three-point bending test, parallel to the layer–interface plane; (**c**) compressive strength test, perpendicular to the layer–interface plane; (**d**) compressive strength test, parallel to the layer–interface plane; (**e**) direct tensile test; (**f**) splitting tensile strength test. F indicates the direction and sense of the forces acting on the specimens during the respective strength tests.

As part of the experimental program, a series of tests were performed on specimens produced using 3D printing technology (Figure 4).

The scope of the investigation included the determination of the following:

#### 2.1.1. Flexural Strength

- Perpendicular to the layer orientation:

A three-point bending test was conducted on the specimens to determine their flexural strength. The procedure was carried out using a testing machine equipped with appropriate tooling. The load was applied at a constant rate of 50 N/s until the specimen failed.

The following formula was used to calculate the flexural strength:

(1) $$\sigma_{max,\;B=}\frac{M_{g,max}}{W_{g}},$$

(2) $$M_{g,max}=\frac{F_{max}\cdot a}{4},$$

(3) $$W_{g}=\frac{bh^{2}}{6},$$

where

$\sigma_{max,B}$—maximum bending stress, (MPa);
$M_{g,max}$—maximum bending moment, (Nm);
$W_{g}$—section modulus for bending, (m^3^);
$F_{max}$—maximum failure load, $\left(kN\right);$
a—span between supports, (m);
b—width of the cross-section, (m);
h—height of the cross-section, (m).

- Parallel to the layer orientation:

The second variant of the flexural strength test involved measuring this parameter with the printed layers oriented parallel to the direction of the applied force. To ensure a flat and even surface, the lateral irregularities of the specimens—caused by the layer deposition process—were filled with leveling mortar. The mortar was applied one day before testing. This approach allowed the test to be conducted properly without increasing the strength of the specimens, as the mortar had not yet developed its full mechanical properties. To determine the flexural strength, the same Equations (1)–(3) were used as in the test of specimens oriented perpendicular to the layers.

#### 2.1.2. Compressive Strength

- Perpendicular to the layer orientation:

During the compressive strength tests, the load was applied to the specimen at a rate of 500 N/s until failure occurred. The head of the testing machine includes a swivel joint, allowing it to compensate for any minor misalignments or non-parallel surfaces. 

The following Equation (4) was used to calculate the compressive strength; for the surface area, the minimum cross-section was determined for the narrowest cross-section:

(4) $$\sigma_{max,C=}\frac{F_{max}}{S}$$

where

$\sigma_{max,C}$—maximum stress in compression, compressive strength, (MPa);
$F_{max}$—load at failure, (N);
S—minimum cross-section determined for the narrowest cross-section, (${mm}^{2})$.

- Parallel to the layer orientation:

The second variant of the compressive strength test involved measuring specimens with the printed layers oriented parallel to the direction of the applied force. As in the flexural strength specimens, mortar was used to level the lateral surfaces of the samples. To determine the compressive strength, the same Equation (4) was used as in the test of specimens oriented perpendicular to the layers.

#### 2.1.3. Direct Tensile Strength

The tensile strength test was conducted on 3D-printed specimens. To determine the tensile strength, the direct tensile method was applied, in which the applied force acts perpendicular to the printed layers. Steel end caps with a diameter of 5 cm were bonded to the ground surfaces of the specimens using a two-component adhesive (POXIPOL). The diagram of the test with bonded surfaces is shown in Figure 4e.

The load was applied at a rate of 100 N/s until failure occurred. Hinged joints in the upper and lower grips ensured uniform load distribution and minimized undesired stress concentrations. This testing methodology allows for determining the maximum tensile stresses the material can withstand under a static direct tensile load. At the same time, it provides insight into the adhesion between printed layers under direct tensile conditions.

The following Equation (5) was used to calculate the direct tensile strength; for the surface area, the minimum cross-section was determined for the narrowest cross-section:

(5) $$\sigma_{max,\;D=}\frac{F_{max}}{S}$$

where

$\sigma_{max,D}$—maximum stress in compression, compressive strength, (MPa);
$F_{max}$—load at failure, (N);
S—minimum cross-section determined for the narrowest cross-section, (${mm}^{2})$.

#### 2.1.4. Splitting Tensile Strength

The splitting tensile strength test was conducted on 3D-printed specimens. After appropriate preparation, the specimens were placed in the testing machine and loaded using cylindrical bearing strips (Figure 4f). The load was applied at a rate of 50 N/s until failure occurred.

This method was adopted from the concrete splitting tensile strength test for prisms (Brazilian method) [40]. The following formula was used to calculate the splitting strength:

(6) $${\sigma_{max,S}}_{=}\frac{{2\cdot F}_{max}}{\pi \cdot d\cdot L}$$

where

$\sigma_{max,S}$—maximum stress in splitting, splitting tensile strength, (MPa);
$F_{max}$—maximum load, (N);
d—sample width, (mm);
L—sample length, (mm).

To establish a reference level for flexural and compressive strength (tests conducted according to the procedure illustrated in Figure 4), measurements were conducted on cast specimens made from the same material used in the 3D printing process, from which the printed samples were also produced. To determine the flexural strength values, Equations (1)–(3) were used, while Equation (4) was applied for compressive strength.

**Figure 5:**
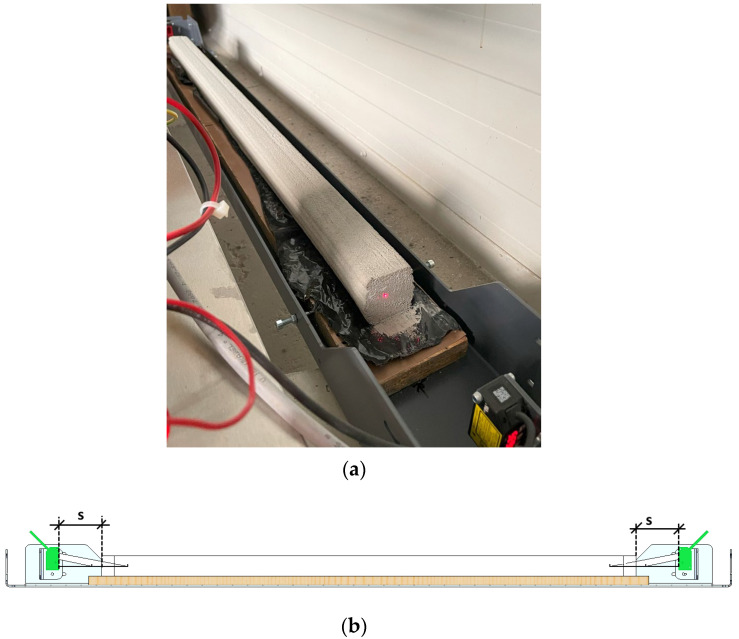
Shrinkage test of 3D-printed materials: (**a**) testing stand for a shrinkage test; (**b**) the test scheme.

In addition to strength testing, the material used was subjected to shrinkage verification during the setting and early curing stages. For this purpose, specimens with dimensions of 40 mm × 40 mm × 1000 mm were printed. The measurements were carried out using a device (Figure 5) equipped with two laser sensors that recorded the change in distance (S), thereby indicating the change in the specimen’s length.

To verify the influence of the time interval between successive printed layers on the anisotropy of mechanical properties and the quality of interlayer bonding, a Portland cement-based material was used. This material was selected for its suitable pumpability, buildability, and open time, tailored to the available printing system. In addition to material characteristics, other influencing factors include the design of individual system components—such as the mixing station, which was developed within the framework of an “Implementation Doctorate” and tested under industrial conditions in partner companies—as well as processing parameters.

Material specimens for testing were produced using both 3D concrete printing (3DCP) and conventional casting methods (in accordance with EN 196-1 [41]), allowing for the assessment of the printing process’s impact on the final properties of the elements. All specimens were prepared in a laboratory environment equipped for testing 3D printing technologies.

**Figure 6:**
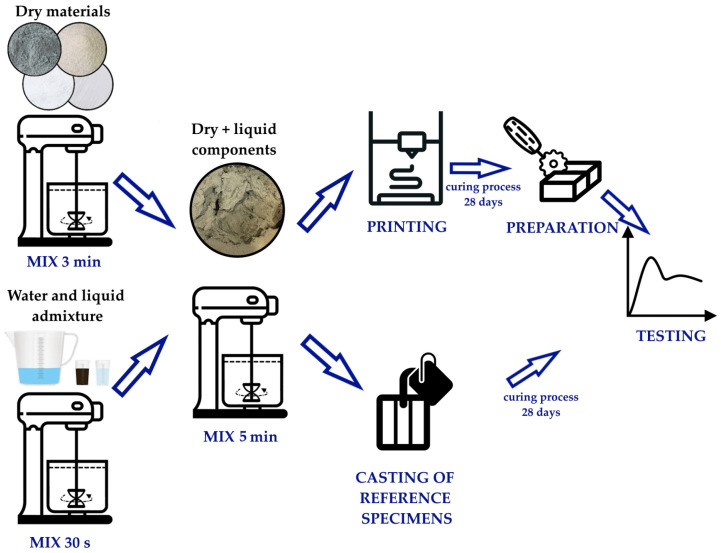
Schematic of the specimen preparation procedure for mechanical testing.

Planetary mortar mixer with a capacity of 20 L was used to prepare the test material, and the detailed mixing procedure is illustrated in Figure 6. Initially, dry and liquid components were mixed separately in individual containers for 3 min and 30 s, respectively. The dry components were then added to the liquid mixture and mixed for an additional 5 min until a homogeneous material was obtained. The prepared mixture was simultaneously poured into molds and placed into the print head.

After 24 h, the cast specimens were demolded, and the printed samples were transferred from the printing platform to a curing chamber. Following a 28-day curing period under controlled conditions of 22 ± 2 °C, the printed specimens were trimmed to the required dimensions. Subsequently, all samples were subjected to mechanical strength testing.

### 2.2. Materials

The specimens for testing interlayer cohesion were produced using a Portland cement-based material, which served as the base composition derived from prior 3D printing trials.

The sample was prepared using a base mix, the composition of which is presented in Table 1. The formulation should ensure not only the achievement of the required design strength but also appropriate rheological and processing properties, which are critical in 3D printing technology. In its fresh state, the mixture must exhibit the ability for stable and continuous extrusion through the print head, without the clogging or segregation of components, while also maintaining the intended geometry after layer deposition. At the same time, the material should possess sufficient structural integrity to allow for the stacking of successive layers without deformation or collapse. Strong interlayer adhesion is also of key importance, as it is essential to ensure structural uniformity and proper load transfer within the printed element.

To meet these requirements, the mix was modified by incorporating a range of additives and admixtures. The composition included agents that accelerate early concrete setting (accelerators), aimed at shortening the time required to reach initial strength and increasing the stabilization speed of successive layers. Rheological additives, such as plasticizers, were also used to improve the workability of the mix while maintaining appropriate viscosity and structural stability. Mineral fillers, including ground limestone and microsilica, were introduced to modify the cement matrix structure, both by enhancing cohesion and increasing the specific surface area, which contributes to improved control over the setting and hardening processes.

### 2.3. Mixture Preparation and Consistency Verification

A laboratory 3D printer and a precision laboratory scale (accuracy of 0.1 g and maximum capacity of 20 kg) were used to prepare the test mixtures. The dry components were first weighed and thoroughly mixed. Water was then added using a 1:5 *w*/*w* (weight-to-weight) ratio, and the mixing was performed with a laboratory planetary mixer under constant conditions: 5 min of mixing at 150 RPM. The resulting mixtures were subjected to consistency testing prior to printing.

**Figure 7:**
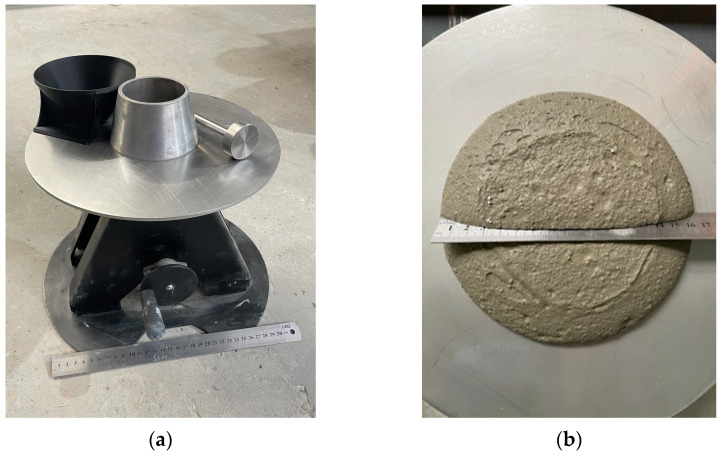
(**a**) View of the flow table test device according to the PN EN 1015-3 standard; (**b**) material sample at consistency test.

To evaluate the consistency of the fresh material intended for printing, a flow table test was conducted in accordance with EN 1015-3 [42]. The device was operated manually (Figure 7). The standard cone was filled with the test material following a two-stage compaction procedure. The test commenced upon removal of the cone and consisted of lifting the flow table 15 times using a handwheel.

Proper consistency is essential not only for maintaining continuous material flow during printing but also for preserving the intended shape of the printed element [9,19,43]. The 3D printing device used in this study is equipped with a screw-based extruder capable of processing materials with a consistency range of 120–200 mm, as defined by EN 1015-3. This range ensures proper extrusion performance and shape retention of the printed object.

**Figure 8:**
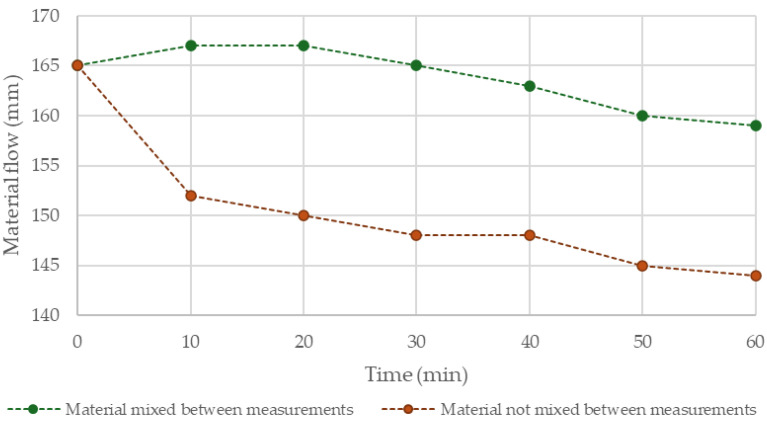
Variation in material flow over time depending on continuous mixing.

In all tests conducted within the framework of this study, fixed proportions of dry components to water were used, resulting in a flow diameter of 165 mm. This is a desirable value, ensuring stable extrusion and sufficient structural integrity of the material after printing. Any change in the consistency of the mix directly affects the extrusion efficiency and may influence the geometry and quality of the printed components [19]. The rheological properties of the tested mix are illustrated in Figure 8, which presents the change in flow diameter over time from the moment of mixing. A comparison was made between mixtures left at rest and those subjected to continuous mixing.

The material, when continuously mixed, maintains a stable consistency over an extended period, limiting the risk of reduced extrusion efficiency, nozzle blockage, and thus deformation or deterioration of the print quality. The initial increase in flow diameter may be attributed to the thorough distribution of components during the first minute of mixing. The subsequent mild decline in flow indicates a gradual onset of setting processes, yet without a sudden loss of workability. In contrast, the material not subjected to re-mixing (e.g., after extrusion) shows a significant drop in flow diameter within the first minute after mixing is stopped. This loss of workability hinders proper material shaping and may lead to discontinuities, lack of interlayer adhesion, or heterogeneity in the interfacial zones. On the other hand, this behavior is also considered desirable, as it positively contributes to the stability of printed elements and their capacity to bear the load of subsequent layers.

### 2.4. Printing

**Figure 9:**
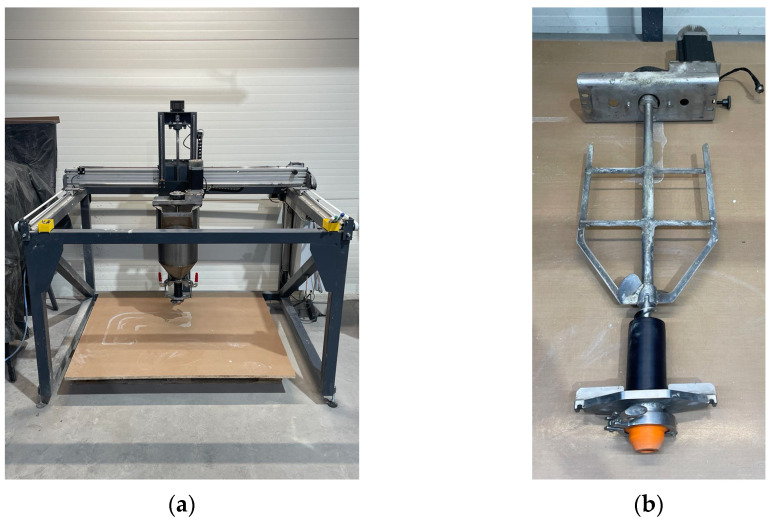
Laboratory setup for 3D concrete printing: (**a**) printer with printhead; (**b**) interior of the printhead with mixer and extruder.

Printed specimens were produced using a laboratory testing setup consisting of a 3D printer with a build volume of 1200 mm × 550 mm × 400 mm. The printer is equipped with a print head that includes a 20 L hopper with an integrated mixer and a screw-based extruder (Figure 9).

**Figure 10:**
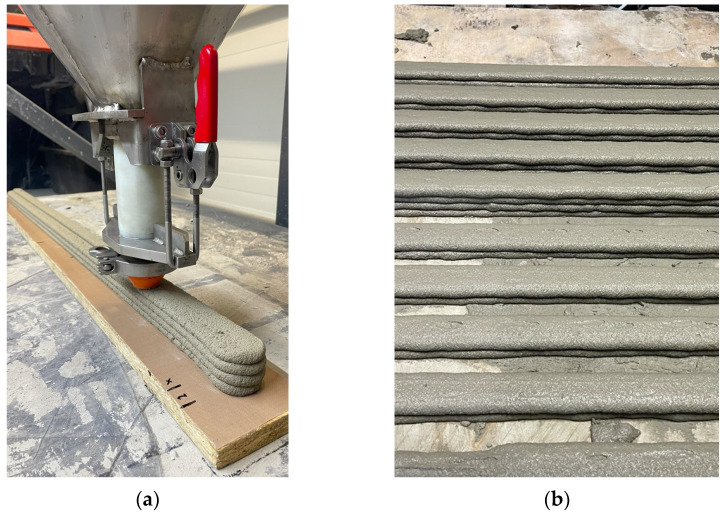
Sample printing: (**a**) printed specimen on a base for material shrinkage testing; (**b**) printed specimens for strength testing.

To investigate the influence of the time interval on the quality of interlayer bonding in printed elements, specimens were prepared in the following three variants (Figure 10):

- Variant 0—no significant time gap between the deposition of successive layers;
- Variant 25—25 min interval between successive layers;
- Variant 50—50 min interval between successive layers.

### 2.5. Preparation

After printing, the specimens were subjected to a curing process. In accordance with standards for cement-based materials, the curing period was set at 28 days, after which the specimens reached their final strength.

Material preparation also included trimming the elements to the required dimensions in a consistent manner for each specimen. A table saw was used for this purpose.

**Figure 11:**
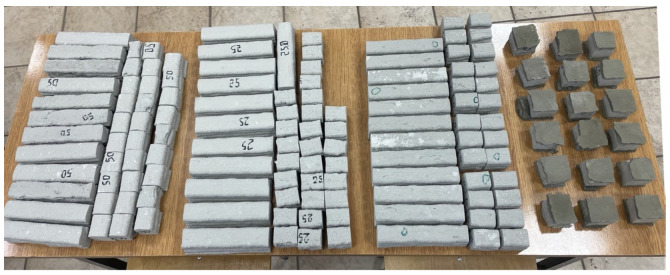
Printed specimens prepared for testing.

The final step in specimen preparation involved ensuring a smooth and flat surface on which the load would act during testing. A grinding stone was used for this purpose, providing a simple and effective means of achieving the required surface quality. For specimens tested parallel to the layer orientation, a thin leveling layer of cement mortar was applied in a 1:4 w/c (water-to-cement) ratio (Figure 11). This leveling layer was applied one day prior to testing to ensure that the added material would not affect the measurement results of the base material.

The tests were carried out on specimens cut from a larger printed element and trimmed to dimensions of approximately 40 mm × 40 mm × 40 mm. For the flexural strength tests, specimens measuring 40 mm × 40 mm × 160 mm were prepared. The cutting was performed in such a way that each specimen included four layers of the printed material. Surface grinding was applied to ensure flatness and uniform load distribution across the entire surface of the specimen. Each mechanical test was conducted on a set of six specimens.

### 2.6. Reference Samples

**Figure 12:**
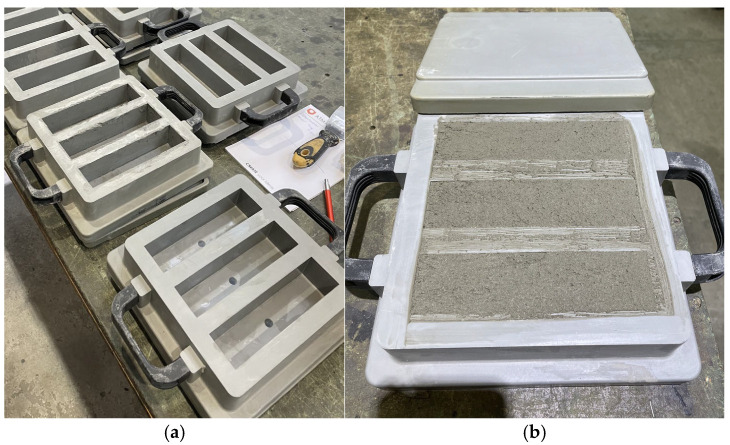
Preparation of specimens using the casting method: (**a**) three-part molds compliant with EN 196-1 standards; (**b**) mold filled with material.

As a control test, measurements were performed on specimens produced using the casting method. Three-part molds were used to fabricate the samples, allowing for the preparation of three specimens with dimensions of 40 mm × 40 mm × 160 mm (Figure 12).

As part of the control testing on cast specimens, the same material was used, and identical proportions of dry components to water were applied as in the 3D printing process. The material was cast into three-part molds intended for the preparation of test specimens in accordance with EN 196-1 standards.

After casting, the specimens were covered and subjected to a curing process for a period of 28 days. Following curing, the material was tested for flexural strength, followed by compressive strength testing.

The printed elements were evaluated in terms of density using the hydrostatic weighing method. This technique enables the determination of the density of objects with complex geometry through simple measurement tools, based on Archimedes’ principle.

The average density of cast specimens was 1969 kg/m^3^. For the specimens produced using 3D printing technology, an average density of 1919 kg/m^3^ was obtained, which is approximately 2.5% lower than that of the reference cast samples. No significant differences in density were observed between the individual variants of the printed specimens.

The lower density of the printed samples may be attributed to the presence of micropores and interlayer gaps resulting from the layer-by-layer deposition of material without additional mechanical compaction. This finding is consistent with trends reported in the literature, indicating that additively manufactured components tend to exhibit slightly higher porosity compared to their conventionally cast counterparts.

### 2.7. Testing

The tests were carried out using a ZwickRoell 50 kN testing machine (ZwickRoell GmbH & Co. KG, Ulm, Germany), which enables the performance of compression, tension, shear, and splitting tests.

The testing machine was equipped with the necessary tools designed for conducting the respective tests and operated using testXpert III software (version 1.2).

## 3. Results and Discussion

### 3.1. Bending Test

**Figure 13:**
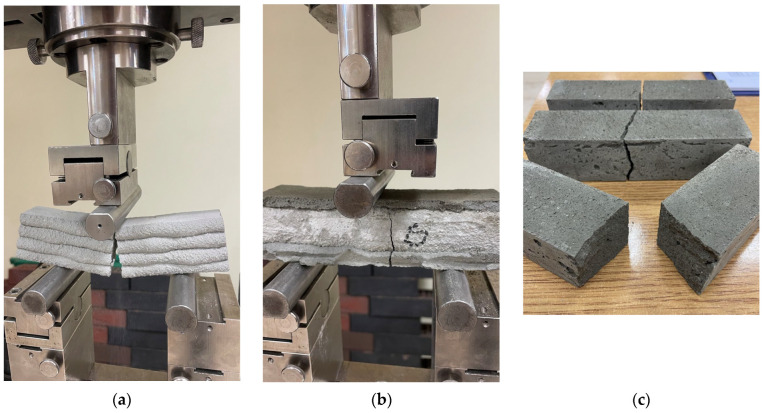
The failure mode of 3D-printed materials after the bending test: (**a**) perpendicular to the layer; (**b**) parallel to the layer; (**c**) mold cast specimens.

The flexural strength test was conducted using cylindrical supports along with a testing apparatus capable of adjusting to the inclination angle of the specimen surfaces (Figure 13). The printed specimens tested perpendicular to the layer orientation, as well as the cast specimens, consistently fractured at the point of load application, precisely between the two supports (Figure 13a,c). In contrast, for the printed specimens tested parallel to the layers, the fracture frequently occurred slightly offset from the point of force application (Figure 13b).

**Figure 14:**
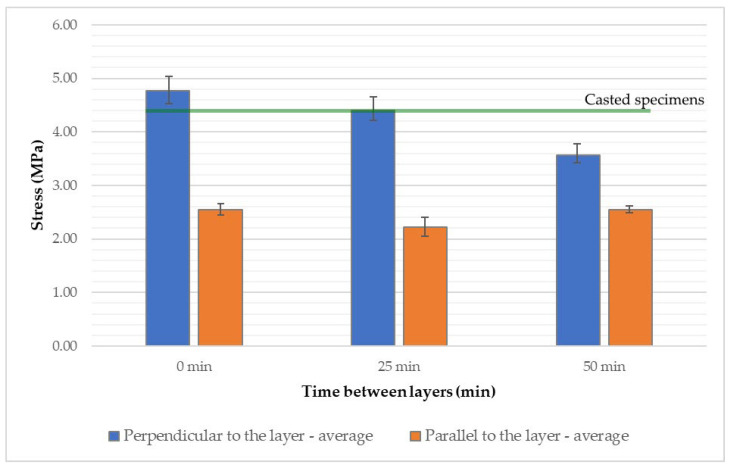
Flexural strength of 3D-printed concrete specimens depending on the applied time interval between successive printed layers.

Analyzing the results of the three-point bending tests on specimens loaded perpendicular to the printed layers (Figure 14 and Supplementary Materials Tables S1–S3), it can be observed that increasing the time interval between layers from 0 to 50 min leads to a noticeable decrease in flexural strength. The average stress values drop from approximately 4.8 MPa to around 3.5 MPa. The extended time gap between layer depositions weakens the interlayer bond, thereby reducing structural integrity. As the layers do not fully integrate, the vertical cohesion of the structure is compromised.

The flexural strength in the direction parallel to the printed layers remains nearly constant regardless of the time interval between layer depositions, with average values ranging between 2.2 and 2.5 MPa. In this orientation, the strength primarily depends on the properties of the concrete mixture itself and the extrusion process, while the time gap between layers, and thus the quality of interlayer adhesion, has a less significant impact.

Referring to the results of the cast specimens, which showed an average flexural strength of 4.34 MPa, it can be concluded that the printing process had only a minor effect on the structural strength when tested perpendicular to the layers. In fact, the printed specimens without a time interval exhibited values up to 10% higher, which is a surprising result. This may be attributed to insufficient compaction in the cast samples or inaccuracies in the printed specimen dimensions caused by uneven side surfaces.

However, the anisotropy in flexural strength is clearly confirmed by the results of printed specimens tested parallel to the layers, where values were approximately 60% lower compared to both the cast specimens and those printed and tested perpendicular to the layers.

### 3.2. Compressive Test

**Figure 15:**
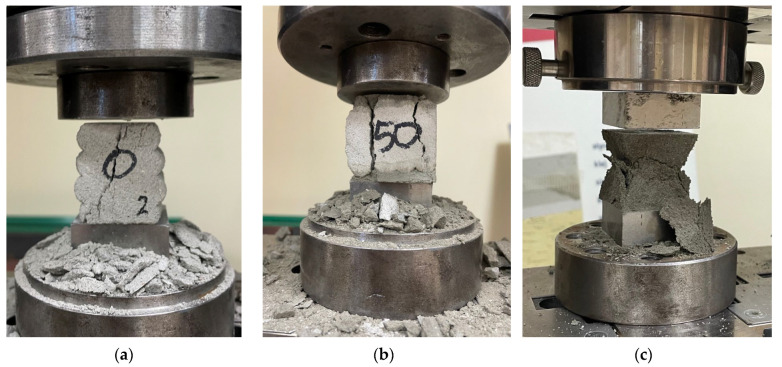
The failure mode of 3D-printed materials after the compressive test: (**a**) perpendicular to the layer; (**b**) parallel to the layer; (**c**) mold cast specimens.

Compressive strength was determined by placing the specimens between two steel platens and applying a load (Figure 15). The 3D-printed specimens tested perpendicular to the layer orientation exhibited relatively random failure patterns, with cracks propagating in various directions across the specimen surface (Figure 15a). The printed specimens tested parallel to the layers predominantly failed along the interlayer interfaces (Figure 15b). In contrast, the cast specimens displayed a typical failure mode for this type of test, characterized by the formation of compression cones (Figure 15c).

**Figure 16:**
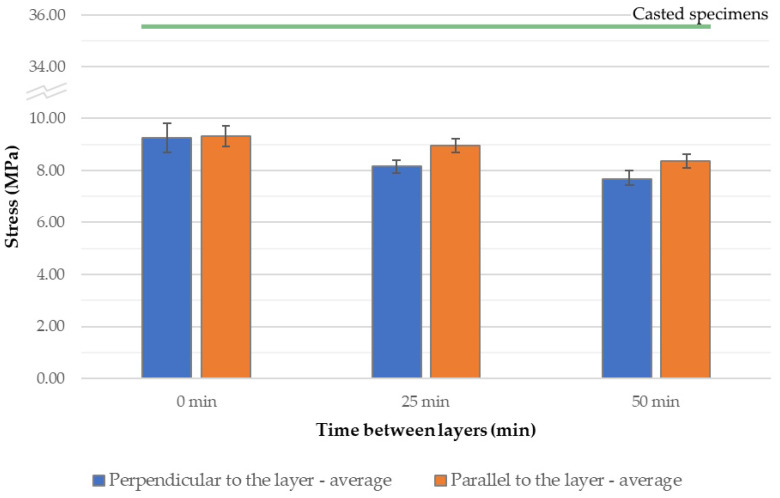
Compressive strength of 3D-printed concrete specimens depending on the applied time interval between successive printed layers.

The compressive strength of the tested material in the direction perpendicular to the layers ranged from 7.65 to 9.24 MPa, with the highest average strength observed in specimens printed without a time interval between layers. Similarly, the compressive strength in the direction parallel to the layers ranged from 8.36 to 9.31 MPa, and again, the highest value of 9.31 MPa was recorded for specimens printed without a time interval. Based on the results obtained in this study, a clear advantage in the strength of cast specimens can be observed. This may be attributed to significantly better compaction of the material during the casting process compared to 3D printing. Additionally, the printed specimens exhibit numerous constrictions, which contribute to the weakening of the overall structure. Compressive strength also shows a decreasing trend with increasing time intervals between successive printed layers (Figure 16 and Supplementary Materials Tables S4–S6). In this type of test, both perpendicular and parallel specimens exhibited a comparable reduction in strength when a 50 min interval was introduced—approximately 17% and 10%, respectively. It is worth noting that, similar to the flexural strength tests, specimens tested parallel to the layers demonstrated less sensitivity to extended time gaps. In this case, however, they achieved slightly higher compressive strength values compared to the specimens tested perpendicular to the layers.

In the case of compressive strength testing, the printing process had a significant impact on the results, as the cast specimens achieved values three to four times higher than those of the printed ones. This is characteristic of 3D concrete printing technology, where the lack of full structural cohesion, the presence of pores, and weakened interlayer zones reduce the material’s load-bearing capacity.

### 3.3. Direct Tensile Strength and Splitting Test

**Figure 17:**
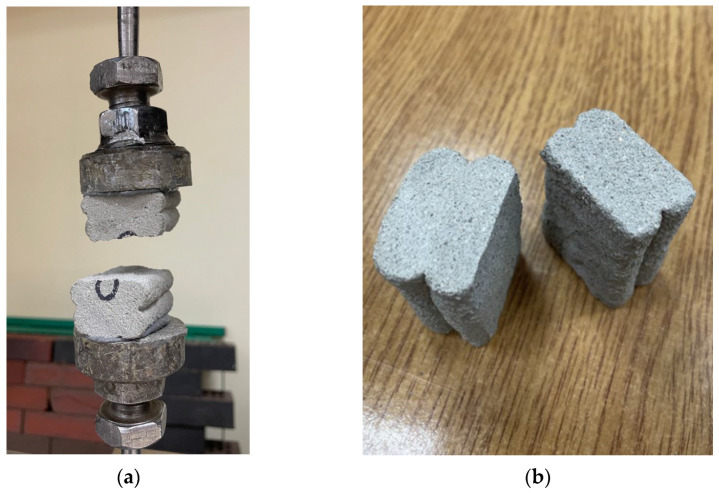
The failure mode of 3D-printed materials after: (**a**) direct tensile test; (**b**) splitting test.

The direct tensile tests (Figure 17a) and the splitting tests (Figure 17b) proceeded as expected, with specimen failure occurring along the printed layer interface, i.e., the weakest zone of the specimen, where cross-sectional narrowing and deformations resulting from interlayer bonding are present.

The tensile strength of the tested material ranged from 0.35 to 1.49 MPa. The highest average strength was achieved by the specimens printed without a time interval between layers, at 1.31 MPa. Notably, among the specimens produced with time intervals, those with a 50 min gap performed better. This may be attributed to slight differences in the cross-sectional area of the samples; in the case of specimens printed with shorter intervals, the cross-section was significantly smaller due to printing parameter inconsistencies.

The obtained tensile strength results indicate a high degree of variability in this property. The standard deviation was 0.38 MPa for the specimens with a 25 min interval and 0.32 MPa for those with a 50 min interval. Failure consistently occurred at the interlayer interface, where the cross-sectional area of the specimen was smallest.

As with the previous tests, the highest strength was observed in the specimens printed without a time interval between layers. The average splitting tensile strength of the material was 1.26 MPa. The results showed considerable variation—when measured along the direction of the printed layers, the standard deviation was 0.27 MPa. All tested specimens failed at the interlayer interfaces, as shown in the photograph in Figure 17b.

**Figure 18:**
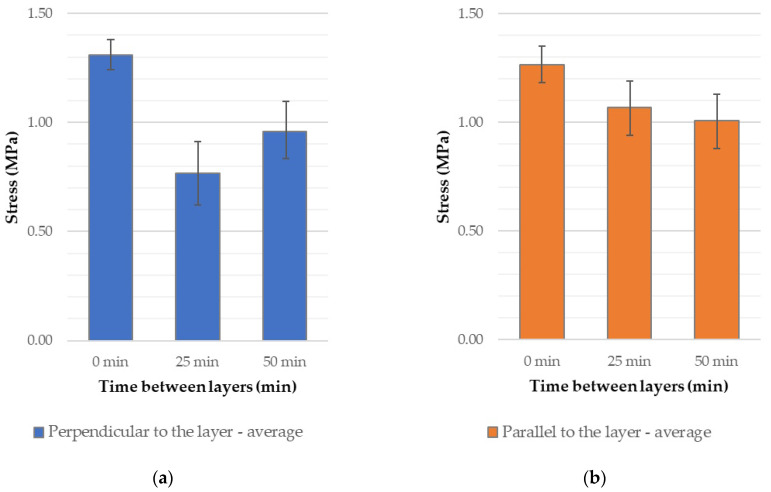
Tensile strength of 3D-printed concrete specimens depending on the applied time interval between successive printed layers: (**a**) direct tensile method; (**b**) splitting method.

Tensile strength, measured using the direct method in the direction perpendicular to the layers, clearly highlights the weakness of the interlayer bonds. Extending the time interval between successive layers leads to a reduction in adhesion, resulting in a significant decrease in strength (Figure 18, and Supplementary Materials Tables S7 and S8). The minimum strength—41% lower than the baseline (no time gap)—was recorded for the 25 min interval, which may correspond to a critical point of adhesion loss due to the previous layer’s surface becoming too dry before the next layer was applied. Additionally, the high sensitivity of the direct tensile test method to defects and cross-sectional irregularities in printed structures contributes to the observed strength reduction. This sensitivity is further confirmed by the large variability in the test results.

In the case of the splitting tensile test, the average strength decreased over time, from a baseline value of approximately 1.26 MPa at 0 min to about 1.01 MPa at 50 min, representing a reduction of around 20%. The strength decline was moderate and more uniform compared to the direct tensile test.

### 3.4. Shrinkage

**Figure 19:**
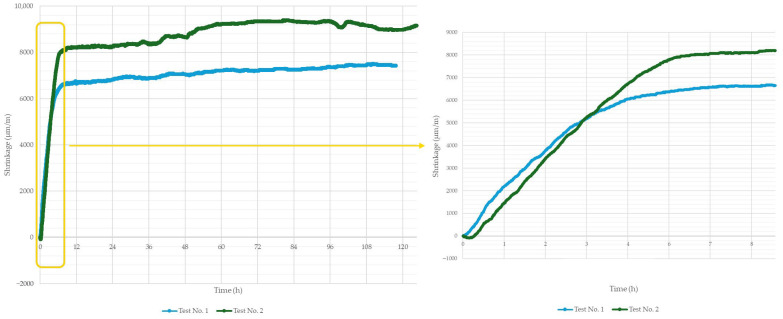
Evolution of total shrinkage over time for 3D-printed samples, with a detailed view of the first 8 h after printing.

As part of the conducted tests, the shrinkage behavior of the printing material was also evaluated. For this purpose, a printed specimen with dimensions of 1000 mm × 40 mm × 40 mm was prepared and placed on a low-friction base. Dimensional changes resulting from shrinkage were recorded by two oppositely positioned laser sensors. The recorded shrinkage over time is presented in Figure 19.

The observed total shrinkage reached unusually high values for a material based on Portland cement [44,45]. This significant volumetric change may be attributed to the rapid setting of the material, accompanied by considerable heat generation. Additionally, shrinkage tends to intensify under conditions that promote moisture loss. The average air temperature and relative humidity during testing were 22.6 °C and 42% for Test 1, and 22.7 °C and 41.2% for Test 2.

A particularly rapid shrinkage rate was observed during the early setting and curing phase, especially within the first 6 h after printing. Within just the first hour, shrinkage values of approximately 1500–2000 μm/m were recorded, which may negatively affect the quality of interlayer bonding when longer time intervals between printed layers are used.

**Figure 20:**
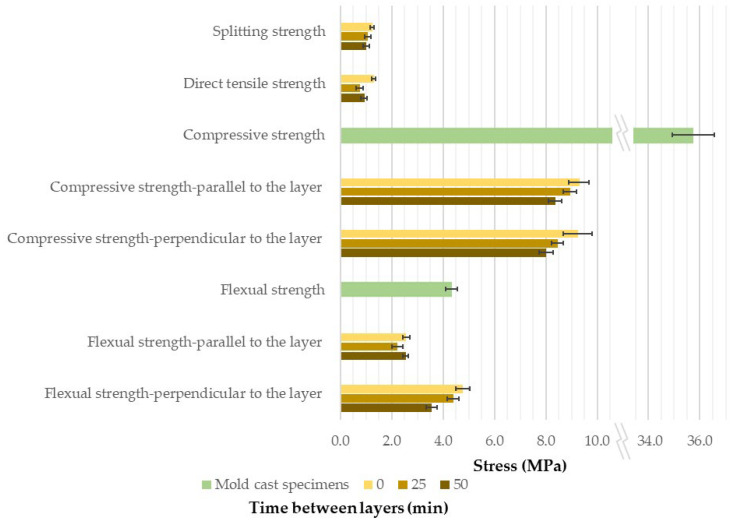
Summary of the tests conducted to determine the mechanical properties of the printed specimens.

Based on the results from all tests conducted to evaluate anisotropy and interlayer cohesion in printed specimens, it can be observed that, in most cases, the highest strength values were recorded for specimens printed without any time interval between layers (Figure 20). This confirms the correlation between the time gap during printing and the quality of interlayer bonding.

The obtained results are fully consistent with the findings reported by other researchers in the field. Babafemi et al. [39] and Miah et al. [46] demonstrated a clear dependency between interlayer bond strength and the time interval between layer depositions in 3D concrete printing. Their studies confirmed that prolonged delays lead to reduced cohesion between successive layers, thereby diminishing the overall interlayer bond strength. Similarly, Moelich, Kruger, and Combrinck [47] introduced the concept of Maximum Operational Time (MOT), defined as the maximum allowable interval that ensures sufficient interlayer adhesion. Their findings indicated that exceeding this time threshold results in a weakened interlayer interface, which aligns well with the trends observed in the present study. Ghosh et al. [48] further reported a notable decrease in flexural strength with increasing time intervals of 30 and 60 min, which they attributed to the loss of mix fluidity retention over time. The most recent comprehensive review by Mishra et al. [49] concluded that extended interlayer pauses contribute to increased porosity, degraded interfacial microstructure, and reduced flexural performance. However, the incorporation of admixtures such as fibers or hydration retarders was found to mitigate these adverse effects to a certain extent. For instance, Pan and Jiang [31] demonstrated that the use of a fluidity-retaining polycarboxylate-based superplasticizer (FR PC) effectively mitigates the reduction in interlayer bond strength associated with extended time intervals between successive layer depositions, specifically within the range of 20 s to 30 min. This finding confirms that both the time interval between layer applications and the composition of the concrete mix are critical parameters for controlling the quality and integrity of the interlayer bond in 3D concrete printing.

**Figure 21:**
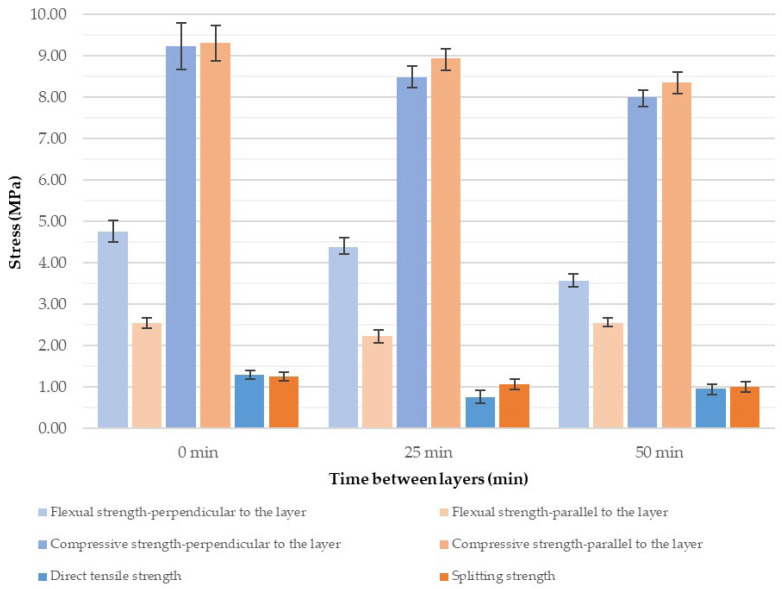
Summary of the tests conducted to determine the mechanical properties of the printed specimens, with color-coded assignment corresponding to the testing direction.

The diagram in Figure 21 highlights the anisotropic nature of 3D-printed concrete specimens, where strength measured perpendicular to the layers is highly dependent on the time interval between successive layers. This indicates a degradation of interlayer bonding over time. In contrast, parallel testing directions are less sensitive, as they rely more on the continuity of the material rather than on adhesion between layers. Flexural strength exhibits the most pronounced anisotropy, making it a critical indicator for assessing structural cohesion in practical applications of 3D concrete printing (3DCP).

When comparing the test results of printed and cast specimens, a significant difference in compressive strength is evident. In the case of cast samples, the strength is approximately 3–4 times higher than that of the printed ones. This further confirms the influence of both the quality of the interlayer bonding, which can weaken the printed element, and the accuracy of the cross-sectional geometry. Numerous constrictions observed in printed specimens may negatively affect their load-bearing capacity.

When analyzing the impact of time intervals between successive layers on the mechanical performance of printed elements, it is also important to consider the effect of material shrinkage. As the material sets and cures over time, shrinkage occurs, altering the geometry of the printed structure. The conducted tests showed that a time gap of approximately 1 h can reduce the length of a 1 m specimen by as much as 2 mm. Such delays between layers can result in cumulative layer misalignment, which may, in turn, compromise the stability and strength of the final structure.

**Figure 22:**
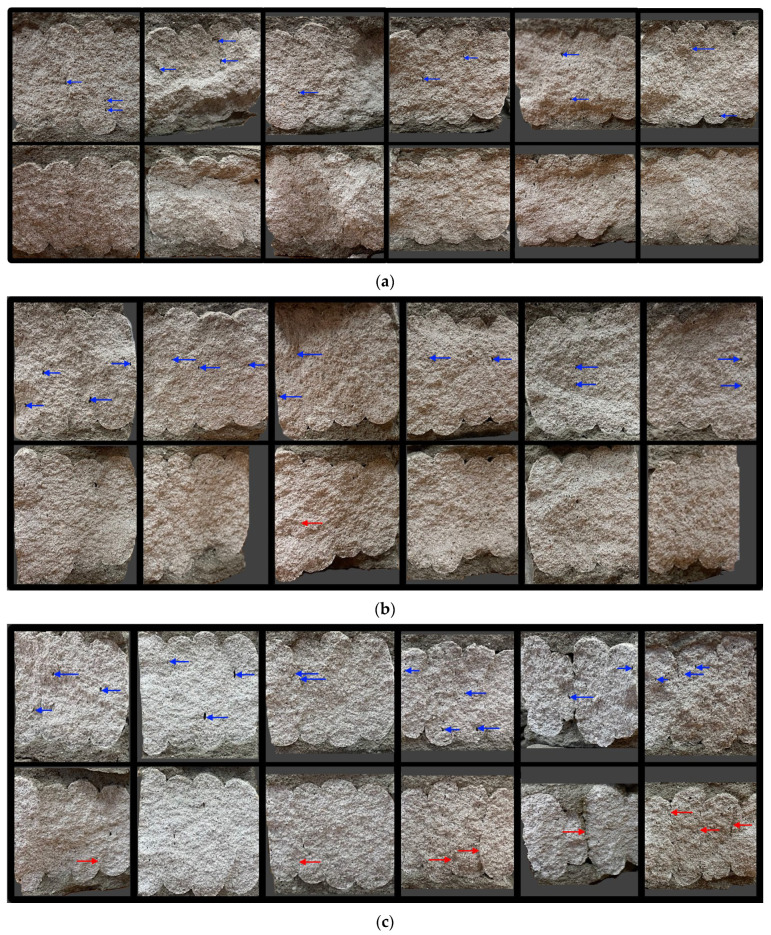
Internal structure of printed specimens: (**a**) without a time interval; (**b**) with a 25 min interval; (**c**) with a 50 min interval between layers. Cross-sections obtained after compressive strength testing in the direction parallel to the printed layers. Blue arrows on the upper cross-sections of each series indicate the presence of pores, while red arrows on the lower cross-sections highlight discontinuities at the interlayer interfaces.

Structural analysis was carried out on the printed specimens subjected to flexural testing in the direction parallel to the printed layers. The obtained cross-sections are presented in Figure 22. Internal pores observed within the structure are marked with a blue indicator, while visible material discontinuities at the interlayer boundaries are marked with a red indicator.

Visual analysis of the internal structure of the printed specimens, presented in Figure 22, reveals a clear influence of the time interval between layers on the integrity of the material. Specimens printed without a delay exhibit a relatively compact and continuous structure, with few visible pores (indicated by blue arrows) and no major discontinuities along the interlayer zones. Introducing a 25 min interval results in a greater number of internal pores and the emergence of weak bonding areas at the interfaces. In the case of a 50 min delay, both the quantity and size of interlayer discontinuities increase significantly, as indicated by the presence of red arrows pointing to cracks and separations at the layer boundaries. This progressive deterioration of the interfacial regions correlates with the observed reduction in compressive strength and confirms that prolonged pauses between layers adversely affect interlayer adhesion and overall structural integrity. These findings underscore the importance of maintaining continuous material deposition in 3D concrete printing processes.

## 4. Conclusions

Three-dimensional concrete printing technology is a rapidly evolving field of materials and mechanical engineering, gaining increasing application within the construction industry. This article presents an overview of the current state of knowledge regarding the mechanical properties of mineral-based materials used in 3D printing for structural purposes.

The primary objective of the research was to evaluate the impact of time intervals between the deposition of successive layers on the mechanical properties and anisotropy of printed elements. Printing process parameters have a direct influence on the quality of interlayer bonding, which plays a crucial role in the load-bearing capacity of elements produced using 3D concrete printing (3DCP), as interlayer connections often constitute the weakest zones within the material structure.

To comprehensively assess the mechanical behavior of printed specimens, tests were conducted to determine flexural, compressive, and tensile strength using both direct tensile and splitting methods. Based on the obtained results, the following conclusions were drawn:

- Extending the time interval between layers in 3D concrete printing (3DCP) has a negative effect on flexural strength in the direction perpendicular to the layers. A 50 min interval between successive layers resulted in a 25% reduction in strength compared to samples printed without interruption due to weakened interlayer bonding. In contrast, time gaps had no significant impact on strength in the parallel direction; however, the testing orientation itself led to a 60% reduction in strength compared to both perpendicular and cast specimens.
- Increasing the time interval between layers also caused a decrease in compressive strength in both directions—perpendicular and parallel to the printed layers. This effect was more pronounced in the perpendicular orientation, underscoring the importance of maintaining continuous layer deposition in 3DCP technology.
- Despite the use of the same material, the forming method significantly influenced the final strength of the concrete. Printed samples (3DCP) achieved only about 25–30% of the compressive strength of cast specimens. However, the flexural strength of cast specimens was comparable to that of printed specimens tested perpendicular to the layers. Nevertheless, further work is needed to improve interlayer adhesion and to optimize 3D printing parameters.
- Direct tensile testing proved more sensitive to the quality of interlayer bonding, highlighting the most critical weaknesses in 3DCP structures. In contrast, splitting tensile tests primarily reflect the general material properties and microstructure, and are somewhat less influenced by printing parameters. In both methods, extended interlayer time intervals negatively impacted tensile strength, though to varying degrees.
- Compressive and flexural strength measured perpendicular to the layers showed a strong dependency on the time interval between layers, indicating degradation of interlayer bonding. Strength in the parallel direction was less affected, as it depends more on material continuity than on adhesion. Flexural strength was found to be the most anisotropic parameter.
- Structural analysis of the cross-sections showed that extending the time interval between layers results in an increased number of pores and the formation of discontinuities at the interlayer interfaces. The observed defects indicate a weakening of interlayer adhesion and a deterioration of the structural cohesion of the printed material.
- In cases where extended time intervals between layers are unavoidable, particular attention should be paid to the shrinkage behavior of the printing material. Significant volumetric changes during setting and early curing may lead to geometric distortions and, consequently, to the reduced stability and strength of 3D-printed structures.

In the context of materials engineering and 3D concrete construction, optimizing the time interval between layer depositions is critical to ensuring structural quality and safety. The interlayer time gap is a key design parameter in 3D-printed concrete structures and should be minimized, particularly when high strength is required in the direction perpendicular to the printed layers.

## Figures and Tables

**Table 1:** The mixture composition of reference mix used for 3D printing tests [19].

Component	Content (%)
Portland cement CEM I 52,5R	25.0
Quartz sand (0.1–1.0 mm)	65.0
Activating additives	2.0
Rheological additives	1.0
Mix fillers	7.0

## Data Availability

The original contributions presented in this study are included in the article and Supplementary Materials. Further inquiries can be directed to the corresponding authors.

## Supplementary Materials

The following supporting information can be downloaded at: https://www.mdpi.com/article/10.3390/ma18163845/s1, Table S1: The results of the flexural strength perpendicular to the layer–interface plane. Table S2: The results of the flexural strength parallel to the layer–interface plane. Table S3: The results of the flexural strength of mold cast specimens. Table S4: The results of the compressive strength of 3D-printed materials, perpendicular to the layer. Table S5: The results of the compressive strength of 3D-printed materials, parallel to the layer. Table S6: The results of the compressive strength of mold cast specimens. Table S7: The results of the direct tensile strength of 3D printed specimens. Table S8: The results of the splitting strength of 3D printed specimens.
