# Supplementary material for: Anisotropy of Mechanical Properties of 3D-Printed Materials—Influence of Application Time of Subsequent Layers
Source: Materials (Basel). 2025 Aug 15;18(16):3845. doi: 10.3390/ma18163845 (PMC12387291; doi:10.3390/ma18163845)
Supplement: Supplementary file 1 [file materials-18-03845-s001.zip › materials-3763203-supplementary.pdf]

Supplementary Materials

Table S1. The results of the flexural strength perpendicular to the layer–interface plane.

Time Between Layers	No	Height *h*	Width *b*	Support Span *a*	F_{max}	$\sigma_{max,B}$	Average
(min)	-	(mm)	(mm)	(mm)	(N)	(MPa)	(MPa)
0	1	41.7	26.5	100	1540	5.01	**4.76**
	2	42.2	25.1		1130	3.79	
	3	41.1	28.0		1800	5.71	
	4	42.7	24.2		1370	4.66	
	5	41.6	27.0		1560	5.01	
	6	41.8	26.6		1360	4.39	
25	1	37.0	36.0	100	1410	4.29	**4.39**
	2	35.5	37.5		1240	3.94	
	3	36.6	36.5		1470	4.51	
	4	34.5	36.8		1340	4.60	
	5	36.0	36.7		1190	3.75	
	6	37.0	34.5		1660	5.27	
50	1	41.0	29.6	100	1190	3.59	**3.56**
	2	36.8	33.8		1020	3.35	
	3	41.7	32.5		1320	3.50	
	4	40.3	30.8		1040	3.12	
	5	39.0	31.5		1390	4.35	
	6	36.5	33.3		1020	3.45	

Table S2. The results of the flexural strength parallel to the layer–interface plane.

Time Between Layers	No	Height *h*	Width *b*	Support Span *a*	F_{max}	$\sigma_{max,B}$	Average
(min)	-	(mm)	(mm)	(mm)	(N)	(MPa)	(MPa)
0	1	35.2	42.6	100	889	2.53	**2.55**
	2	35.6	41.1		922	2.66	
	3	35.9	40.9		808	2.30	
	4	35.6	41.4		826	2.36	
	5	35.5	41.4		1030	2.97	
	6	36.0	40.6		872	2.49	
25	1	35.1	47.7	100	967	2.48	**2.23**
	2	35.5	45.6		969	2.54	
	3	35.5	45.0		857	2.27	
	4	33.4	47.0		804	2.30	
	5	35.0	47.5		600	1.55	
50	1	33.6	41.3	100	779	2.51	**2.55**
	2	32.7	40.3		761	2.66	
	3	35.1	41.2		843	2.50	
	4	36.4	46.3		1130	2.77	
	5	35.3	45.5		980	2.60	
	6	35.2	46.9		867	2.24	

Table S3. The results of the flexural strength of mold cast specimens.

No	Height h	Width b	Support Span a	F_{max}	$\sigma_{max,B}$	Average
-	(mm)	(mm)	(mm)	(N)	(MPa)	(MPa)
1	40.0	40.0	100	2060	4.83	**4.34**
2	40.0	40.0		2190	5.13	
3	40.0	40.0		1530	3.59	
4	40.0	40.0		1710	4.01	
5	40.0	40.0		1820	4.27	
6	40.0	40.0		1800	4.22	

Table S4. The results of the compressive strength of 3D-printed materials, perpendicular to the layer.

Time Between Layers	No	Cross-Section S	F_{max}	$\sigma_{max,C}$	Average
(min)	-	(mm²)	(N)	(MPa)	(MPa)
0	1	1196.3	9450	7.90	**9.24**
	2	1076.1	10,600	9.85	
	3	1168.5	11,900	10.18	
	4	962.5	10,800	11.22	
	5	1040.0	8720	8.38	
	6	1147.0	9080	7.92	
25	1	1125.8	8600	7.64	**8.15**
	2	1139.5	9700	8.51	
	3	1104.6	8260	7.48	
	4	982.3	8040	8.18	
	5	1078.7	8750	8.11	
	6	994.5	8900	8.95	
50	1	1194.8	8390	7.02	**7.65**
	2	1191.0	9550	8.02	
	3	1136.3	9600	8.45	
	4	1052.1	7970	7.58	
	5	1100.8	7920	7.19	

Table S5. The results of the compressive strength of 3D-printed materials, parallel to the layer.

Time Between Layers	No	Cross-Section S	F_{max}	$\sigma_{max,C}$	Average
(min)	-	(mm²)	(N)	(MPa)	(MPa)
0	1	1600.0*	15,700	9.81	**9.31**
	2		14,700	9.19	
	3		12,600	7.88	
	4		15,700	9.81	
	5		13,700	8.56	
	6		17,000	10.63	
25	1	1600.0*	14,900	9.31	**8.95**
	2		14,600	9.13	
	3		13,300	8.31	
	4		12,900	8.06	
	5		14,000	8.75	
	6		16,200	10.13	

50	1	1600.0*	12,300	7.69	**8.36**
	2		12,900	8.06	
	3		15,200	9.50	
	4		14,400	9.00	
	5		12,600	7.88	
	6		12,900	8.06	

* Pressure surface according to the dimensions of the testing machine tool- 40 × 40 mm^2

Table S6. The results of the compressive strength of mold cast specimens.

No	Cross-Section S	F_{max}	$\sigma_{max,C}$	Average
**-**	**(mm^2)**	**(N)**	**(MPa)**	**(MPa)**
1	1600.0*	59,600	37.25	**35.77**
2		63,300	39.56	
3		59,900	37.44	
4		63,000	39.38	
5		58,600	36.63	
6		62,300	38.94	
7		53,300	33.31	
8		53,600	33.50	
9		51,600	32.25	
10		52,700	32.94	
11		54,100	33.81	
12		54,800	34.25	

* Pressure surface according to the dimensions of the testing machine tool – 40 × 40 mm^2

Table S7. The results of the direct tensile strength of 3D printed specimens.

Time Between Layers	No	Cross-Section S	F_{max}	$\sigma_{max,D}$	Average
**(min)**	**-**	**(mm)**	**(N)**	**(MPa)**	**(MPa)**
0	1	1070.65	1370.0	1.28	**1.31**
	2	1196.25	1720.0	1.44	
	3	1177.2	1680.0	1.43	
	4	1292.1	1930.0	1.49	
	5	1291.95	1430.0	1.11	
	6	1196.01	1330.0	1.11	
25	1	1169.1	412.0	0.35	**0.77**
	2	959.14	595.0	0.62	
	3	922.2	699.0	0.76	
	4	1046.4	480.0	0.46	
	5	1166.79	1590.0	1.36	
	6	1280.3	1350.0	1.05	
50	1	1220.9	1420.0	1.16	**0.96**
	2	1307.9	1870.0	1.43	
	3	1328.4	1170.0	0.88	
	4	1152.58	736.0	0.64	
	5	1281.28	781.0	0.61	
	6	1360.8	1400.0	1.03	

Table S8. The results of the splitting strength of 3D printed specimens.

Time Between Layers	No	Length *L*	Width *d*	F_{max}	$\sigma_{max,S}$	Average
(min)	-	(mm)	(mm)	(N)	(MPa)	(MPa)
0	1	44.0	29.5	2770.0	1.36	**1.26**
	2	43.9	30.5	3050.0	1.45	
	3	43.9	30.4	2960.0	1.41	
	4	43.8	29.5	2110.0	1.04	
	5	43.8	29.9	2000.0	0.97	
	6	43.8	30.0	2800.0	1.36	
25	1	43.9	26.5	1250.0	0.68	**1.07**
	2	43.9	29.5	2620.0	1.29	
	3	43.4	27.0	2390.0	1.30	
	4	43.2	30.8	2610.0	1.25	
	5	43.8	22.0	1080.0	0.71	
	6	42.6	29.5	2300.0	1.17	
50	1	42.5	27.0	1490.0	0.83	**1.01**
	2	41.8	32.8	2450.0	1.14	
	3	42.5	28.2	1450.0	0.77	
	4	42.0	33.6	3290.0	1.48	
	5	43.0	28.9	2170.0	1.11	
	6	42.4	25.0	1190.0	0.71	
